# Tobacco use among a population of women attending cervical cancer screening programs in primary health care clinics in South Africa: a cross-sectional study

**DOI:** 10.11604/pamj.2022.43.14.31611

**Published:** 2022-09-08

**Authors:** Christine Njuguna, Joel Msafiri Francis, Olalekan Ayo-Yusuf, Elizabeth Reji, Agetta Jimmy Akii, Samuel Ubabukoh, John Mukuka Musonda, Joyce Sikwese Musonda, John Ndimande, Langalibalele Honey Mabuza, Olufemi Omole

**Affiliations:** 1 Department of Family Medicine and Primary Care, University of the Witwatersrand, Johannesburg, South Africa,; 2 Africa Centre for Tobacco Industry Monitoring and Policy Research (ATIM), Sefako Makgatho Health Sciences University, Pretoria, South Africa,; 3 Department of Family Medicine, University of Free State, Bloemfontein, South Africa,; 4 Division of Family Medicine, Department of Family Medicine and Primary Care, University of Witwatersrand, Johannesburg, South Africa,; 5 Department of Family Medicine and Primary Care, University of Pretoria, South Africa,; 6 Department of Family Medicine and Primary Health Care, Sefako Makgatho Health Sciences University, Pretoria, South Africa

**Keywords:** Cervical cancer screening, primary health care, women, tobacco use, South Africa

## Abstract

**Introduction:**

we determined the prevalence, patterns and factors associated with tobacco use among women presenting for cervical cancer screening in primary health care clinics in Gauteng province, South Africa.

**Methods:**

this study utilized data from an ongoing cross-sectional study commenced in September 2018, in which 749 participants had responded to an interviewer-administered semi-structured questionnaire on socio-demographics, HIV status, tobacco use, family planning methods, sexual and cervical cancer screening behaviours. Data were entered into the web-based research electronic data capture (REDCap). We performed descriptive data analysis and included multivariate logistic regression. We considered a p-value < 0.05 statistically significant.

**Results:**

participants´ median age was 38 years (interquartile range: 31-38) with 43.9% (328) reporting being HIV-positive. The prevalence of ever and current tobacco use were 24.3% (182/749) and 17.1% (128/749) respectively. In multivariable logistic regression, participants who self-identified as racial ethnicity other than Black African and those who were HIV positive and not on antiretroviral treatment, had increased odds of reporting current tobacco use ((adjusted odds ratio (AOR)= 5.6, 95% CI: 3.2-9.8) and (AOR= 8.2, 95% CI: 2.0-34.1) respectively).

**Conclusion:**

current tobacco use is common among women attending cervical cancer screening programs in primary health care clinics in Gauteng Province. Furthermore, study findings suggest the need to integrate tobacco cessation treatments into women´s health and HIV treatment programs.

## Introduction

Tobacco is a significant public health problem worldwide, and currently accounts for 8 million deaths annually [1]. In 2018, an estimated 23.6% of adults 15 years and older used tobacco worldwide; 38.6% of males and 8.5% of women [2]. However, the prevalence of tobacco use is on the decline globally, falling from 33.3% in 2000 to 23.6% in 2018 [2]. This is largely due to most countries adopting the World Health Organization Framework Convention on Tobacco Control (WHO FCTC) treaty that came into effect in 2005 [1]. The WHO FCTC, the first public health treaty aims to promote the implementation of evidence-based tobacco control policies in countries that have signed this treaty [3]. Such policies include but are not limited to the banning of advertising of tobacco products, printing of graphic warning labels on cigarettes packs, banning sale of tobacco products to minors, banning smoking in public spaces and provision of tobacco dependence treatment, amongst others [3].

In line with global trends, the prevalence of tobacco use in South Africa has also declined [4], owing largely to the stringent tobacco control policies such as price and tax hikes, health warnings on tobacco products and banning of advertisement of tobacco products [4,5]. As reported in the 1998 and 2016 South Africa demographic health surveys, the reported prevalence of daily or occasional smoking has decreased from 11% to 8% among women, and from 42% to 37% in men [5,6]. Smokeless tobacco use has decreased from 11% to 6% among women and remained unchanged in men at 1% [5,6]. However, the prevalence of smoking is still high among the colored/mixed ancestry population (40% among women and 51% among men) [5], raising public health concerns.

Tobacco smoking is associated with an increased risk of morbidity and mortality, particularly for cardiovascular diseases, chronic obstructive lung diseases and cancers [7]. Amongst women, smokers are more likely to develop cervical cancer, the second commonest cancer among women in South Africa [8].

Women presenting to cervical cancer screening programs constitute an at-risk population and therefore a priority group among whom preventative strategies such as delivering tobacco cessation counselling and nicotine dependence treatments and strengthening of HIV prevention and treatment are clinical and public health imperatives. To be effective, these strategies need to be informed by local data on tobacco use and its sociodemographic and clinical correlates originating from these programs. However, data on tobacco use patterns and the associated sociodemographic and clinical factors (such as HIV status, CD4 count and antiretroviral therapy (ART) use) among women attending cervical cancer screening programs is not available in South Africa in particular and it is limited in general. The aim of this study was to determine the prevalence, patterns, and sociodemographic and clinical factors associated with reported tobacco use among women attending cervical cancer screening programs in 20 primary health care facilities in Gauteng Province, the most populous province hosting the economic capital in South Africa.

## Methods

**Study design:** this was a cross-sectional study involving adult women presenting for cervical screening at 20 clinics in Gauteng Province from September 2018 to September 2019.

**Study setting:** we conducted the study in primary health care (PHC) clinics in all the five health districts in Gauteng Province, three metropolitan health districts (City of Johannesburg, Ekurhuleni, and City of Tshwane) and two municipal health districts (Sedibeng and West Rand). The surveyed clinics provide ambulatory comprehensive primary care services: preventative, curative and rehabilitation.

**Study participants and sampling:** all women who presented to the 20 PHC clinics in Gauteng Province for routine pap smears during the study period were eligible to participate in the study. According to South Africa district health barometer, an estimated 155,851 women presented to all the PHC facilities in Gauteng Province for cervical cancer screening during 2013/14 [9]. Considering annual growth in patient numbers of 5%, the estimated number of pap smears performed in Gauteng Province in 2018/19 was estimated at 198,909. Assuming a 50% prevalence of tobacco use, a margin of error of 5%, a 95% confidence level the estimated minimum sample size was determined to be 384 to estimate the prevalence of tobacco use among women attending for cervical cancer screening. However, we included data from all the 749 participants that were already recruited at the time of this data analysis in September 2019.

**Data collection:** patients who presented for pap smear daily at each clinic during the study period were eligible to participate. Illiterate patients had the patient information leaflet read and explained to them by a trained research assistant fluent in English and local languages. Patients who accepted to participate in the study were directed to a private room where written informed consent was obtained by the research assistant. The research assistant administered the study questionnaire.

### Study variables

**Outcome variables:** 1) tobacco use: ever tobacco use was defined as the responses to “how will you describe your tobacco use status?”: “currently smoke cigarette”; “smoked in the past”; “currently used snuff”; “used snuff in the past”; “currently chewed tobacco leaves”; “chewed tobacco leaves in the past”. Current tobacco use defined as one or more of “I currently smoke cigarettes”, “I currently use snuff” and “I currently chew tobacco leaves” in response to the question “how would you describe your tobacco use status?”. Smoking defined as a positive response to using combustible tobacco product in any of commercial or hand-rolled cigarette or cigar in the question “which tobacco type(s) have you ever used?”. Snuff use was defined as a “yes” response to home-made or commercially prepared snuff products or snus. Other tobacco use was a “yes” response to using any of “water-pipe/hookah /hubbly-bubble/shisha”. Tobacco leaves use was defined as a “Yes” response to chewing tobacco leaves; 2) secondhand tobacco smoke exposure defined as a “yes” response to “do you live or work where other people smoke regularly around you?”. The options for the place of regular exposure were “at home”/“work”/“other places”.

**Exposure variables:** 1) socio-demographic characteristics: age at last birthday, level of education (“did not attend school”, “grade 1 to 6”, “grade 7 to 12”, “post-high school”), employment status (artisan, pensioner, professional, student, unemployed, others), marital status (divorced, single, married, widowed, separated), ethnicity (self-identification as Black African or colored/mixed ancestry or Indian or White); 2) HIV status was assessed with a participant´s response to the question “what is your HIV status?” (positive or negative or unknown); 3) CD4 count level defined as response to the question “what was your last CD4 count?” documented as cells/microliter. This is routinely documented for all HIV-positive individuals attending the primary healthcare clinic for routine follow-up; 4) antiretroviral therapy status: defined as a response of “yes” or “no” to the question “if positive HIV status, are you on antiretroviral therapy?”.

**Data management and analysis:** we entered data from paper questionnaires into REDCap. We exported the data from REDCap to STATA 15 (Statacorp, College Station, Texas) for analysis. We used descriptive statistics to describe the participants´ sociodemographic, HIV status, and tobacco use characteristics. We described age using median and interquartile range (IQR). We described categorical variables using simple proportions and conducted bivariate analysis with exposure variables including age, education level, ethnic group (race), employment status, marital status, HIV status, CD4 count and use of ART. Age variable was forced into the model regardless of the p-value at bivariate analysis because it is a known potential confounder, other exposure variables were included in the model if they had a p-value <0.2 in the bivariate analysis, into the multivariable logistic regression models. We reported adjusted odds ratios, their corresponding 95% confidence intervals and p-values. A p-value <0.05 was considered statistically significant.

**Ethical considerations:** the study received ethics approval from University of the Witwatersrand Medical Research Ethics Committee (M160209). All study participants provided written informed consent prior to administration of the e-questionnaire. All participants who responded positively to current tobacco use and other risky behaviors (e.g. unprotected sex or multiple sexual partners or women living with HIV who had not initiated ART) were briefly counseled and assisted to access usual health care services that addressed identified risky behaviors.

## Results

**Participants´ general characteristics:** the study recruited 749 women who had pap smears done and who completed the tobacco survey. Overall, study participants had median age of 38 years IQR (31-38). Of these (n=749) and there were 377 (50.4%) who reported HIV-negative status, 328 (43.9%) who reported living with HIV and 43 (5.7%) who reported unknown HIV status. We also noted that most were single 431 (57.5%), completed high school education 570 (76.8%), unemployed 465 (62.4%) and majority 672 (89.7%) self-identified as Black African (Table 1).

**Table 1:** characteristics of women (N=749) attending pap smears at primary health care clinics in Gauteng Province, South Africa (September 2018-September 2019)

Characteristic	Responses	N (%)
**Age (years)**	**Median [IQR]**	**38 [31-38]**
Age categories (years)^a^	15-24	57 (7.7%)
	25-49	536 (72.0%)
	≥50	151 (20.3%)
**Education^b^**		
	Did not attend school	17 (2.3%)
	Primary school	92 (12.4%)
	High school	570 (76.8%)
	Post-high school	63 (8.5%)
**Employment status^c^**		
	Unemployed	465 (62.4%)
	Employed	246 (33.0%)
	Student	26 (3.5%)
	Others	8 (1.1%)
**Ethnic group**		
	Black African	672 (89.7%)
	Colored	68 (9.1%)
	Other (Whites and Indians)	9 (1.2%)
**Marital status**		
	Single	431 (57.5%)
	Married	188 (25.1%)
	Divorced/separated	130 (17.4%)
**HIV status^d^**		
	Positive	328 (43.9%)
	Negative	377 (50.4%)
	Unknown	43 (5.7%)
**On antiretroviral therapy^e^**		
	Yes	316 (97.2%)
	No	9 (2.8%)
**ART duration (years)^f^**	**Median [IQR]**	**0.6 [0.25-1.0]**
**CD4 count (cells/μl)^g^**		
	<=200	32 (16.5%)
	>200	162 (83.5%)

a : excludes 5 women with missing information on age; ^b^: excludes 7 women with missing education; ^c^: excludes 4 women with missing employment status; ^d^: excludes 1 woman with missing HIV status; ^e^: restricted to HIV positive women only (n=328), excludes 3 women with missing ART; ^f^: excludes 23 women with missing ART duration; ^g^: excludes 134 HIV positive women with missing CD4 count

**Tobacco use characteristics:** among the study participants, 182 (24.3%) participants reported ever tobacco use and 128 (17.1%) reported current tobacco use. Among the current tobacco users, we reported that 63 (49.2%) and 61 (47.7%) smoked cigarette and used snuff exclusively. Only a handful of women reported dual use of tobacco. Overall, the prevalence of current cigarette smoking, and current snuff use were 8.4% (63/749) and 8.1% (61/749), respectively. Among participants who reported current cigarette smoking, most (n=37) (55.2%) reported smoking ≤5 cigarettes per day. Amongst snuff users (n=63), almost half, 23 (41.1%) used snuff 5 or more times, whilst one tobacco leaf chewer used this product 1 to 2 times a day (Table 2).

**Table 2:** tobacco use characteristics of women (N=749) attending pap smears in primary health care clinics in Gauteng Province, South Africa (September 2018-September 2019)

Characteristic	Responses	N (%)
Ever use	Yes	182 (24.3%)
	No	567 (75.7%)
Current use	Yes	128 (17.1%)
	No	621 (82.9%)
**Duration of use for ever tobacco users (n=182)^a^**		
	≤1 year	16 (11.0%)
	1-10 years	75 (51.4%)
	>10 years	55 (37.7%)
**Types of Tobacco products for current tobacco users (n=128)^b^**		
	Cigarettes	63 (49.2%)
	Snuff	61 (47.7%)
	Cigarettes and snuff	2 (1.6%)
	Cigarettes and tobacco leaves	1 (0.8%)
	Cigarettes and water pipe	1 (0.8%)
**Intensity of current tobacco use**		
**Cigarettes (n=67)^c^**		
	5 cigarettes or less per day	37 (55.2%)
	6 to 10 cigarettes per day	23 (34.3%)
	11 to 20 cigarettes per day	5 (7.5%)
	>20 cigarettes per day	2 (3.0%)
**Snuff (n=63)^d^**		
	1 to 2 times per day	16 (28.6%)
	3 to 4 times per day	17 (30.4%)
	5 or more times per day	23 (41.1%)
**Snuff use route (n=63)^e^**		
	Through the nose only	52 (85.3%)
	Through the mouth only	9 (14.8%)
**Quit duration for past tobacco users (n=54)^f^**		
	Less than 6 months	6 (25.0%)
	6 months to 1 year	5 (20.8%)
	1 to 5 years	6 (25.0%)
	>5 years	7 (29.2%)

a : excludes 36 ever tobacco users with missing duration; ^b^: restricted to current tobacco users only (n=128); ^c^: intensity of cigarette users only (n=67); ^d^: intensity of snuff users only-excludes 7 snuff users with missing duration; ^e^: excludes 2 women with missing information on route of snuff use; ^f^: excludes 30 ex-tobacco users with missing information on quit duration

**Factors associated with reported ever tobacco use:** in the multivariable logistic regression model for ever used tobacco, women with post-high school education had lower odds of reporting ever tobacco use compared to women who had never attended school (AOR= 0.3; 95% CI: 0.1-0.9; p=0.037). On the other hand, women who self-identified being of other races (Colored, Indian and White) compared to Black Africans (AOR=2.9; 95% CI: 1.7-5.0; p<0.001), and those living with HIV, not on ART compared to those who were HIV-negative (AOR=4.6; 95% CI: 1.2-18.2; p=0.030), were significantly more likely to report ever use of tobacco (Table 3).

**Table 3:** factors associated with ever tobacco use among women attending pap smears at primary health care facilities in Gauteng Province (September 2018-September 2019)

Variable	Ever tobacco use-yes (n=182)	Ever tobacco use-no (n=567)	Unadjusted odds ratio	95% CI	P value	Adjusted odds ratio	95% CI	P value
**Age categories (years)^a^**								
15-24	13 (22.8%)	44 (77.2%)	Ref			Ref		
25-49	120 (22.4%)	416 (77.6%)	0.9	0.5-1.9	0.943	1.0	0.5-2.0	0.979
≥50	49 (32.5%)	102 (67.6%)	1.6	0.8-3.3	0.177	1.7	0.7-3.7	0.214
**Education^b^**								
Did not attend	6 (35.3%)	11 (64.7%)	Ref			Ref		
Primary school	25 (27.2%)	67 (72.8%)	0.7	0.2-2.0	0.497	0.4	0.1-1.3	0.128
High school	140 (24.6%)	430 (75.4%)	0.6	0.2-1.6	0.318	0.4	0.1-1.3	0.124
Post-high school	9 (14.3%)	54 (85.7%)	0.3	0.1-1.0	0.057	0.3	0.1-0.9	0.037
**Employment status^c^**								
Unemployed	112 (24.1%)	353 (75.9%)	Ref			Ref		
Employed	61 (24.8%)	185 (75.2%)	1.0	0.7-1.5	0.834	-		
Student	8 (30.8%)	18 (69.2%)	1.4	0.6-3.3	0.442	-		
Others	0 (0%)	8 (100.0%)	-					
**Race**								
Black African	147 (21.9%)	525 (78.1%)	Ref			Ref		
Other (Colored, Indian and White)	35 (45.5%)	42 (54.5%)	3.0	1.8-4.8	<0.001	2.9	1.7-5.0	<0.001
**Marital status**								
Single	106 (24.6%)	325 (75.4%)	Ref			Ref		
Married	44 (23.4%)	144 (76.6%)	0.9	0.6-1.4	0.751	-		
Previously married	32 (24.6%)	98 (75.4%)	1.0	0.6-1.6	0.996	-		
**HIV status & antiretroviral therapy (ART) status^d^**								
HIV negative	92 (24.4%)	285 (75.6%)	Ref			Ref		
HIV positive & on ART	72 (22.8%)	244 (77.2%)	0.9	0.6-1.3	0.618	1.2	0.8-1.7	0.424
HIV positive not on ART	5 (55.6%)	4 (44.4%)	3.9	1.0-14.7	0.047	4.6	1.2-18.2	0.030
**CD4 count^e^**								
≤200	11 (34.4%)	21 (65.6%)	Ref			Ref		
>200	34 (21.0%)	128 (79.0%)	0.5	0.2-1.2	0.105	-	-	

a : excludes 5 women with missing age; ^b^: excludes 7 women with missing education; ^c^: excludes 4 women with missing employment status; ^d^: excludes women with unknown HIV status (n=43), women with missing HIV status (n=1) and HIV positive women with missing ART status (n=3); ^e^: restricted to HIV positive women n=328-not included into the final model, 40% (134/328) of CD4 counts missing

**Factors associated with reported current tobacco use:** in multivariable logistic regression current tobacco use was significantly associated with self-identifying as Colored or White or Indian (AOR= 5.6; 95%CI: 3.2-9.8; p <0.001) compared to women of Black African ethnicity, and with women living with HIV not on ART (AOR=8.2; 95% CI: 2.0-34.1; p=0.004) compared to HIV-negative women (Table 4).

**Table 4:** factors associated with current tobacco use among women attending pap smears at primary health care facilities in Gauteng Province (September 2018-September 2019)

Variable	Current smoker-yes (n=128)	Current smoker- no (n=621)	Unadjusted odds ratio	95% CI	P value	Adjusted odds ratio	95% CI	P value
**Age categories (years)^a^**								
15-24	8 (14.0%)	49 (86.0%)	Ref			Ref		
25-49	88 (16.4%)	448 (83.6%)	1.2	0.6-2.6	0.643	1.2	0.5-2.9	0.704
≥50	32 (21.2%)	119 (78.8%)	1.6	0.7-3.8	0.246	1.7	0.6-4.5	0.294
**Education^b^**								
Did not attend school	4 (23.5%)	13 (76.5%)	Ref			Ref		
Primary school	19 (20.7%)	73 (79.4%)	0.8	0.2-2.9	0.790	-		
High school	95 (16.7%)	475 (83.3%)	0.6	0.2-2.0	0.460	-		
Post-high school	8 (12.7%)	55 (87.3%)	0.5	0.1-1.8	0.275	-		
**Employment status^c^**								
Unemployed	81 (17.4%)	384 (82.6%)	Ref			Ref		
Employed	42 (17.1%)	204 (82.9%)	1.0	0.6-1.5	0.908	-		
Student	5 (19.2%)	21 (80.8%)	1.1	0.4-3.1	0.813	-		
Others	0 (0%)	8 (100.0%)	-					
**Race**								
Black	95 (14.1%)	577 (85.9%)	Ref			Ref		
Others (Colored, Indian and White)	33 (42.9%)	44 (57.1%)	4.6	2.8-7.5	<0.001	5.6	3.2-9.8	<0.001
**Marital status**								
Single	77 (17.9%)	354 (82.1%)	Ref			Ref		
Married	28 (14.9%)	160 (85.1%)	0.8	0.5-1.3	0.366	-		
Previously married	23 (17.7%)	107 (82.3%)	1.0	0.6-1.7	0.964	-		
**HIV status and antiretroviral therapy (ART) status^d^**								
HIV negative	61 (16.2%)	316 (83.8%)	Ref			Ref		
HIV positive and on ART	51 (16.2%)	265 (83.9%)	1.0	0.7-1.5	0.988	1.6	1.0-2.5	0.057
HIV positive and not on ART	5 (55.6%)	4 (44.4%)	6.5	1.7-24.8	0.006	8.2	2.0-34.1	0.004
**CD4 count (cells/ul)^e^**								
≤200	10 (31.3%)	22 (68.8%)	Ref			Ref		
>200	24 (14.8%)	138 (85.2%)	0.4	0.2-0.9	0.029	-		

a : excludes 5 women with missing age; ^b^: excludes 7 women with missing education; ^c^: excludes 4 women with missing employment status; ^d^: excludes women with unknown HIV status (n=43), women with missing HIV status (n=1) and HIV positive women with missing ART status (n=3); ^e^: restricted to HIV positive women n=328- not included into the final model, 40% (134/328) of CD4 counts missing

## Discussion

This study found that cigarettes and smokeless tobacco are common among women presenting for cervical cancer screening in primary health care facilities in Gauteng Province and that women living with HIV who were not on antiretroviral treatment (ART) and those who self-identified as Colored, Indian or White ethnicity had significantly higher odds of reporting ever or current tobacco use. In contrast, women with higher education (post-high school) had lower odds of reporting ever tobacco use only. Our findings align with findings of previous South African studies [4,5], but more importantly highlight the need to prioritize and integrate tobacco cessation treatment across all health programs in PHC clinics, including women´s health and HIV treatment programs.

The overall prevalence of reported current tobacco use, smoking, and snuff use in the current study were higher than the national averages reported for women in the most recent South African demographic and health survey (12.6%, 8% and 6% respectively) [5]. In addition, they are higher than rates reported by researchers in other sub-Saharan African studies, where prevalence of cigarettes smoking and snuff use ranged from 0.1% to 3% amongst women [1,10,11]. The higher prevalence of tobacco use in this study may suggest that women who present to cervical cancer screening program in primary care are particularly at higher risk of tobacco attributable diseases than the general women population in South Africa and therefore need to be assisted to quit in order to mitigate this risk.

The patterns and intensity of reported tobacco use observed in this study are consistent with a previous national survey and indicate that most South African women are light cigarette smokers (CPD ≤10) and also use snuff mainly through the nose, mostly <5 times per day [5]. These levels of tobacco use intensities support the notion that most South African tobacco users have low nicotine dependence [12], and may be amenable to non-pharmacological tobacco cessation treatments. However, it was noted that only 29.6% of ever tobacco users reported having quit tobacco use. Our finding, coupled with the high prevalence of current tobacco use further underscores the importance of introducing tobacco cessation interventions targeted at women attending PHC clinics, particularly in women´s health and HIV treatment programs.

This associations found between reported tobacco use and self-identified race group other than Black African, and reporting post-high school educational attainment, is consistent with those of previous South African studies [4-6,13]. Our study findings further reiterate the need to pay attention to racial/ethnic disparities in tobacco use and hence the design of a culturally appropriate tobacco cessation treatments in South Africa. Efforts should be made to ensure that women from these at-higher-risk of tobacco use groups are not missed for targeted preventive interventions. Our finding that participants with higher educational attainment have lower odds of ever tobacco use is consistent with international literature, in that this group is less likely to experience “socio-economic disadvantages” and financial stress, and have a smoker at home and more likely to have a higher sense of agency for quitting when they smoke [14]. While all women ought to be screened, those with lower educational attainment (consequently in the lower socioeconomic status) need to be targeted for tobacco use screening and cessation treatment within an integrated women´s health program, particularly because they are also less likely to present for screening and early intervention for cervical cancer [15].

This study showed a higher odds of reported ever and current tobacco use among women who self-reported being HIV-positive, but not on antiretroviral treatment (ART), thereby reaffirming the suggestion that individuals with one unhealthy/undesirable lifestyle are more likely to engage in other unhealthy lifestyle behaviors [12]. These HIV-positive women who are not on ART may therefore be exhibiting harmful health behaviors in general, and this may in turn put them at risk for other diseases, including cervical cancer [7,16]. To this end, healthcare providers in PHC clinics need to use opportunities provided by clinic visits to implement comprehensive and integrated screening for tobacco use, cervical cancer and HIV, including addressing non-initiation and poor adherence to treatments. This is particularly so, considering that almost half of the women (43.9%) in our study reported living with HIV - a condition which increases the risk of cervical cancer on its own. However, it is important to note that our study did not find any association between HIV status and reported current tobacco use, contrary to findings from South African and sub-Sahara African studies that have reported a positive relationship between HIV positive status, smoking and snuff use [17]. The sample used in the sub-Saharan study was much larger (a pooled analysis of 25 demographic and health surveys) compared to our study among a conceivably high-risk women population attending cervical screening program. This may explain the lack of association between HIV status and reported tobacco use in our study.

The findings of our study should be interpreted considering the following strengths and limitations. The main strength of our study is that it targeted a relatively larger population of at-risk women, i.e. those coming for cervical cancer screening. However, our study has some limitations: firstly, data on tobacco use was self-reported, and not verified using objective measures such as urine and saliva nicotine, and breath carbon monoxide levels. This may have introduced some element of information bias due to social desirability and resulted in under-reporting with underestimation of the outcomes. Secondly, our findings may not be generalized to the general population, as the study population consisted of women attending clinics for pap smear. Thirdly, the cross-sectional study design precludes making causal inference concerning some factors presumed predictive of tobacco use. These notwithstanding, this study provides for the first time the pattern of tobacco-use among women potentially at risk for cervical cancer and its relationship with HIV status and ART. The findings from this study could inform intervention design not only in South Africa, but possibly in other countries with similar settings and have high incidence of cervical cancer and HIV.

## Conclusion

Both cigarette smoking and snuff use is common amongst women attending PHC facilities for pap smear. This necessitates routine screening and implementation of tobacco cessation treatments as promoted in other services. Our finding of a higher odds of reporting current tobacco-use among women living with HIV and not on ART, suggests underlying complex lifestyle behavioral problem. There is a need for a comprehensive and integrated prevention and health promotion strategy in PHC settings that should cut across different priority health programs, particularly in women´s health and HIV treatment programs.

### What is known about this topic

- Tobacco is a significant public health problem worldwide;
- The prevalence of tobacco use is on the decline globally;
- Tobacco smoking and smokeless tobacco use is significantly higher among women living with HIV.

### What this study adds

- The prevalence of tobacco products uses among at risk women attending for primary care facilities for cervical cancer screening;
- Highlights the need for integration of tobacco products use reduction interventions in primary care settings.
